# No evidence of spatial representation of age, but “own-age bias” like face processing found in chimpanzees

**DOI:** 10.1007/s10071-021-01564-7

**Published:** 2021-10-02

**Authors:** Yuri Kawaguchi, Masaki Tomonaga, Ikuma Adachi

**Affiliations:** 1 Messerli Research Institute, University of Veterinary Medicine Vienna, Vienna, Austria; 2 Japan Society for the Promotion of Science, Tokyo, Japan; 3 Primate Research Institute, Kyoto University, Inuyama, Japan; 4 Inuama, Japan

**Keywords:** Age perception, Spatial representation, Own-age bias, Chimpanzee, Face recognition

## Abstract

Previous studies have revealed that non-human primates can differentiate the age category of faces. However, the knowledge about age recognition in non-human primates is very limited and whether non-human primates can process facial age information in a similar way to humans is unknown. As humans have an association between time and space (e.g., a person in an earlier life stage to the left and a person in a later life stage to the right), we investigated whether chimpanzees spatially represent conspecifics’ adult and infant faces. Chimpanzees were tested using an identical matching-to-sample task with conspecific adult and infant face stimuli. Two comparison images were presented vertically (Experiment 1) or horizontally (Experiment 2). We analyzed whether the response time was influenced by the position and age category of the target stimuli, but there was no evidence of correspondence between space and adult/infant faces. Thus, evidence of the spatial representation of the age category was not found. However, we did find that the response time was consistently faster when they discriminated between adult faces than when they discriminated between infant faces in both experiments. This result is in line with a series of human face studies that suggest the existence of an “own-age bias.” As far as we know, this is the first report of asymmetric face processing efficiency between infant and adult faces in non-human primates.

**Supplementary Information:**

The online version contains supplementary material available at 10.1007/s10071-021-01564-7.

## Introduction

Faces convey a lot of information to humans, such as age, identity, gender, and emotional states (Bruce and Young 2012; Rhodes et al. 2011). Non-human primates can also extract various information from faces (Adachi and Tomonaga 2017; Leopold and Rhodes 2010; Parr 2011), and this includes identity (Itakura 1992; Parr et al. 2000), species (Wilson and Tomonaga 2018), sex (de Waal and Pokorny 2008; Koba et al. 2009), social rank (Dahl and Adachi 2013), emotional states (Kanazawa 1996; Parr 2003), attentional states (Tomonaga and Imura 2010), and attractiveness (Waitt et al. 2003). However, facial age perception has not been studied in non-human primates until quite recently, even though it is a well-studied topic in human face recognition (e.g., Burt and Perrett 1995; for review Rhodes 2007). Recognizing conspecific’s approximate age, that is, age category is important for social primates as it enables them to behave appropriately around other individuals by changing their behavior based on age (Berry and McArthur 1986). An infant individual should be treated differently from an adult individual by conspecifics, for example, in that they are vulnerable and cannot survive without care from adults. Non-human primates may use various cues such as body size, body movement, vocalization, and odors, but facial cues can also provide reliable information on age.

Some studies have investigated how non-human primates respond to the face stimuli of adult and infant individuals. For example, Koda et al. (2013) examined whether Japanese macaques (*Macaca fuscata*) exhibit an attentional bias for infant faces, which has been reported in humans (Lucion et al. 2017), but they obtained no evidence to support this. Other studies found that non-human primates can differentiate between faces of different age categories (i.e., adult or infant) (Kawaguchi et al. 2019b, 2020). In these studies, chimpanzees (*Pan troglodytes*) (Kawaguchi et al. 2020) and capuchin monkeys (*Sapajus apella*) (Kawaguchi et al. 2019b) were trained to discriminate between the adult and infant faces of conspecifics or humans using a symbolic matching-to-sample task. Both the chimpanzees and capuchin monkeys easily learned to do this, and this ability was generalized to the discrimination of novel stimuli. These studies demonstrated that the sensitivity to age-related facial features is shared by non-human primates and discussed what kind of facial cues the participants seemingly used for such categorizations. However, compared to the accumulation of human research, there are still a limited understanding of the perception of facial age in non-human primates. Although previous studies have found that non-human primates are able to visually differentiate adult faces and infant faces, it is still unknown whether non-human primates extract an age category concept from faces. They may have categorized adult and infant faces just by combining low-level features without recognizing age. Therefore, we examined whether chimpanzees recognize infants and adults in a certain relationship (i.e., time) as humans do by testing spatial mapping of face age in chimpanzees.

As illustrated by the idiom “from the cradle to the grave,” humans recognize that infants and adults exist linearly in a time sequence. In other words, age has the direction and we understand that an infant will not be an infant forever and that an older person was not old when they were born. Moreover, in most cases, when people illustrate human life stages, the infant is depicted on the left, the “middle” age is placed in the middle, and the older person is presented on the right in a horizontal line. This is because we have a mental timeline, and we associate space and time in a certain direction (e.g., earlier is left; later is right) (Fuhrman and Boroditsky 2010; Santiago et al. 2007; Torralbo et al. 2006; Weger and Pratt 2008). For example, Fuhrman and Boroditsky (2010) presented pairs of pictures one after another, and the participants were required to answer whether the second picture showed either an “earlier” or “later” event than the first picture by pressing keys. The stimuli included short (e.g., filling a cup of coffee) and long (e.g., people of different age classes) time periods. English speakers were faster to make earlier judgments when the corresponding key was positioned at the left, while Hebrew speakers had the opposite pattern. Thus, the direction of mental timelines is influenced by cultural factors, such as writing direction. Furthermore, a larger congruency effect was observed when the stimuli depicted a long-time interval. Spatial representation of time is observed horizontally and vertically in some cultures (e.g., Boroditsky 2001). Moreover, the correspondence between the abstract domain and spatial domain is observed not only for time, but also for other abstract domains, including numbers (for a review see Fias and Fischer 2005), social rank (e.g., Schubert 2005), and auditory pitch (e.g., Rusconi et al. 2006). Each abstract domain is mapped horizontally, vertically, or both. One example of vertical spatial representations is social status, and it has been demonstrated that “high-ranked” individuals are represented in spatially higher positions than “low-ranked” individuals by human adults (Schubert 2005).

The correspondence between the abstract domain and space is also observed in non-human animals. For example, there is some evidence of the spatial mapping of numbers in various animals including chicks (Rugani et al. 2015, 2017), rhesus macaques (Drucker and Brannon 2014), and chimpanzees (Adachi 2014), although the direction of spatial mapping may vary within and across species (Johnson-Ulrich and Vonk 2018). Furthermore, Dahl and Adachi (2013) conducted a matching-to-sample task in which chimpanzees were required to discriminate between the face identities of familiar group members that were presented in a vertical arrangement and found that chimpanzees have a spatial mapping of the dominance hierarchy similar to humans. They reported that when the rank of the represented individual and the position in the display were congruent (e.g., a high-ranked individual was positioned higher), the response time was faster than when they were incongruent. These comparative studies suggest that spatial representation have evolutionary roots and emerged before language evolution, while they are also flexible so that their direction can be changed by culture (e.g., Shaki and Fischer 2008). One explanation of such phenomena is that space and other magnitude may be associated in animal brains when they are represented (Rugani and de Hevia 2017).

Given those evidences in non-human animals especially the one showing spatial representation of the social domain (Dahl and Adachi 2013), it is not tested but possible that non-human primates have a particular spatial representation of age as reported in the humans (Fuhrman and Boroditsky 2010). Thus, our main aim of this study was to investigate whether chimpanzees spatially represent conspecifics’ adult and infant faces in order to understand whether they recognize infants and adults in time (or at least any other abstract domain which has a direction). Our prediction was that if chimpanzees refer a conceptual age category that can be recognized in a time sequence from a face, they would respond faster when the spatial arrangement of face stimuli are congruent with their time representation, if any. A previous studies have demonstrated that spatial and time judgments interact in rhesus macaques (Mendez et al. 2011; Merritt et al. 2010). However, as far as we know, no study has investigated the space-based representation of time in non-human primates.

Although testing spatial mapping of face age was the main purpose of this study, we also investigated whether the chimpanzees’ performance in discriminating adult faces and infant faces is asymmetric because face processing is largely modulated by the amount of the experiences. Enhanced experiences of specific face categories in early and late-life selectively tune perceptual systems for face processing toward that category. For example, older infants (9 months) and adults can discriminate among conspecific faces, but not monkey faces, while younger infants (6 months) can discriminate both of them (Pascalis et al. 2002). Such perceptual tuning based on very early experience in life is called perceptual narrowing and is observed in other face categories such as own- versus other-race faces in humans (“own-race bias,” e.g., Kelly et al. 2007). In addition to such early perceptual tuning, later exposure or expertise throughout life also modulates face processing. For example, Koreans living among Caucasians from childhood show identify Caucasian faces better than Asian faces (Sangrigoli and Pallier 2005). Enhanced face processing by extensive exposure in later life also occurs with faces of specific age categories as “own-age bias” (Wright and Stroud 2002). This bias is a phenomenon in which human adults have superior processing for adult faces compared with processing for children’s faces and vice versa. It is considered that such a bias, like other biases in face processing, results from more frequent exposure to individuals from the same age group than to others in daily social life (Rhodes and Anastasi 2012). For example, preschool teachers can recognize children’s faces and adults’ faces equally well (Kuefner et al. 2008).

The enhanced face processing by both early and late exposure of specific face categories has also been reported in non-human primates. Dahl and his colleague investigated captive chimpanzees’ face discrimination ability for both conspecifics and humans (Dahl and Adachi 2013). They found that young chimpanzees with less exposure to humans have advantages in discriminating chimpanzees rather than human faces, while adult chimpanzees with lifelong exposure to humans have advantages with human faces over conspecific faces. However, it remains unknown whether the amount of experience with a specific age category also affects face processing efficiency in non-human primates. Therefore, we compared the performance of adult chimpanzees when they discriminated between adult faces and infant faces to explore whether they also exhibit this age-related asymmetric processing efficiency.

To investigate those two aspects, namely spatial mapping and the amount of exposure related to age, we used a matching-to-sample task in which chimpanzees were required to match the faces of either adult or infant individuals. We applied and modified the procedure of the previous study, which reported the vertical representation of dominance in chimpanzees (Dahl and Adachi 2013). In the matching-to-sample task, two comparison images were presented in vertical (Experiment 1) or horizontal (Experiment 2) arrays. We examined whether their performance differed depending on the correspondence between the position and the age category of the stimuli. To examine the spatial correspondence effect, the two comparison images were from different age categories (i.e., one adult and one infant) in one condition, and they were from the same age category in the other condition. We also compared their discrimination performance for adult faces, and that for infant faces to examine if they have age-related asymmetric processing efficiency based on the different amount of the experiences.

## Methods

### Participants

Six chimpanzees (*Pan troglodytes verus*) living at the Primate Research Institute, Kyoto University, participated in the experiments. All of them were adults (17–41 years old), and one was male (see Table 1 for more individual information). They are living as a social group made up of 11 adult individuals and all of them had experience of interacting with infants before. The chimpanzees live in an enriched environment with an outdoor compound (700 m^2^) and an indoor enclosure. They also have access to a semi-outdoor residence (Matsuzawa 2006). They are neither food- nor water-deprived, and they live in social groups. They receive food several times each day, and they always have access to water.

**Table 1:** Participant information

Individual name (GAIN^1^ ID)	Sex	Age	Birth experience
Ai (0434)	Female	41	Parous
Ayumu (0608)	Male	18	–
Chloe (0441)	Female	37	Parous
Cleo (0609)	Female	18	Nulliparous
Pal (0611)	Female	17	Nulliparous
Pendesa (0095)	Female	41	Nulliparous

^1^GAIN (the Great Ape Information Network) is an information network about Hominoidea living in Japan

The participants were called for the experiments daily, and their participation was voluntary. During the experiment, they were unrestrained, and they could stop the task whenever they wanted to. All of them had abundant experience of matching-to-sample tasks, including in Dahl and Adachi’s previous study. All procedures adhered to institutional guidelines (the Primate Research Institute’s 2010 version of “The Guidelines for the Care and Use of Laboratory Primates”). The experimental design was approved by the Animal Welfare and Animal Care Committee of the Primate Research Institute (2018–115) and the Animal Research Committee of Kyoto University.

### Apparatus

All of the experiments were conducted in an experimental booth (1.8 m wide × 2.15 m deep × 1.75 m high). The participants were tested using touch-sensitive 17-inch LCD monitors (LCD-AD172F2-T monitor, 1280 × 1024 pixels) and universal feeders (BUF-310, Biomedica). The stimuli presentation, touch detection, and reward delivery during the experiments were controlled using personal computers (PC-9821 Xn, NEC Corp.). The experimental program was written in Microsoft Visual Basic 2010 Express (Microsoft Corp.).

### Stimuli

We used six adult and six infant chimpanzee face images as the stimuli. Most of the photographic images were either taken by the author or provided by colleagues, while a few were obtained from public sources. The depicted individuals were unfamiliar to the participants, and they showed neutral expressions. Half of the adult chimpanzees were males, while the sex of some of the infant chimpanzees was unknown. Unfortunately, the exact ages of some of the infants in the images taken from public sources were also unknown. However, we selected pictures of infants who appeared to be younger than two years old when the pictures were taken. Using Adobe Photoshop Elements 15 (Adobe Inc., San Jose, CA, USA), all of the images were cropped into a square with 250 × 250 pixels (6.6 cm × 6.6 cm), their luminance was matched, and they were presented in color.

### Procedure

**Fig. 1:**
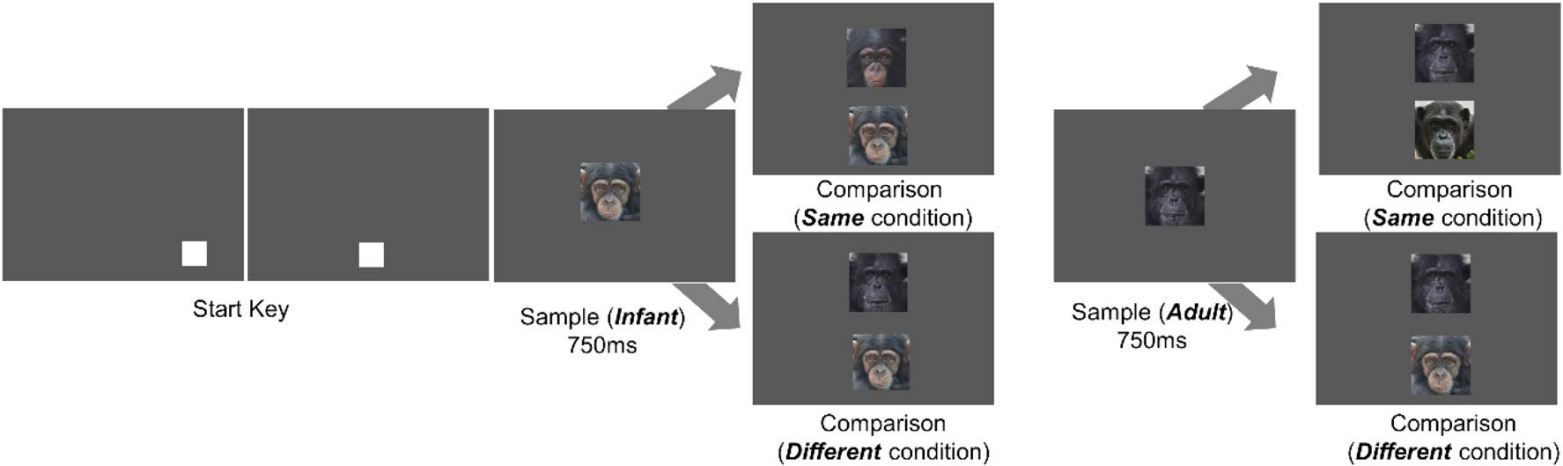
An example of one trial in Experiment 1 (vertical array). The self-start key was presented at the bottom. When the participant touched it, a sample stimulus was presented in the center of the monitor for 750 ms. When the sample disappeared, two comparison images were presented, and the participant was required to touch the same stimulus. In the same condition, the two comparison images were from the same age category, while in the different condition, they were from different age categories

The participants were required to perform an identical zero-delay matching-to-sample task (Fig. 1). Each trial began when the participant touched the self-start key that appeared at the bottom of the monitor after a 2-s inter-trial interval. The self-start key appeared twice in different positions at the bottom of the monitor, with the second one always being presented in the center of the bottom of the monitor. When they touched the start keys, a sample image appeared in the center of the monitor for 750 ms. Two comparison images then appeared, one of which was identical to the sample stimulus. The participants were required to choose the same image. When they chose the correct answer, a piece of apple was delivered via the universal feeder as a reward.

In Experiment 1, the two comparison images were presented in a vertical array, while in Experiment 2, they were presented in a horizontal array. In both experiments, the two comparison images were from the same age category (i.e., both were adults/both were infants) in the same condition, and they were from a different age category (i.e., one was an adult and the other was an infant) in the different condition. In each experiment, there were 66 combinations of the comparison images as there were 12 stimuli in total. For each combination, there were two comparison arrays (top or bottom in Experiment 1/left or right in Experiment 2) and two sample stimuli (either of the comparison images). Hence, the total 264 trials were divided into six sessions. In one session, 20 trials were presented in the same condition, and 24 trials were presented in the different condition. The order of the conditions and stimuli was pseudo-randomized.

### Data analysis

#### Behavioral data analysis

In both experiments, the number of correct responses and the response times to choose the correct answers were analyzed. The accuracy was calculated and arcsine transformed for each condition, and we conducted a 2 × 2 × 2 ANOVA of the position (top or bottom/left or right), age of the stimuli (adult or infant), and condition (same or different) as the independent variables. For the response time, only the response times of the correct trials were analyzed. We excluded response times that were longer than the average value plus three standard deviations (*SDs*) as the chimpanzees were sometimes distracted by unexpected noise from outside or by something else during the experiment and took longer to respond. The response time was analyzed using a 2 × 2 × 2 ANOVA with the same independent variables as the analysis of the accuracy. All the statistics were conducted by R 4.1.0 (R Core Team 2018).

#### Image analysis

When a different performance of the discrimination between adult and infant faces was found, this asymmetry may be caused by the variation in the physical characteristics of the infant faces just being smaller than that of the adult faces. To compare the physical variation in the face stimuli within each age category, we conducted an image similarity analysis of the stimuli and compared it between the age categories. The similarity between each exemplar (adult faces [*n* = 6] and infant faces [*n* = 6]) was evaluated for all combinations within the same age category. We used the structural similarity index (“SSIM,” Wang et al. 2004), which is widely used to measure the similarity of two images by comparing local patterns of pixel intensity. The analysis was conducted using Python (Python Software Foundation, Wilmington, DE, USA) and OpenCV (Intel Corp., Santa Clara, CA, USA). All stimuli were converted to grayscale, and the SSIM was calculated for all of the possible combinations. The SSIM could range from – 1 to + 1, and if the two images were identical, the score was 1. To calculate the physical distance between each of the stimuli, this SSIM score was subtracted from 1. The calculated differential score between every stimulus combination within each age category was compared using the Mann–Whitney U-test.

## Results

### Behavioral data

#### Experiment 1 (vertical array)

**Fig. 2:**
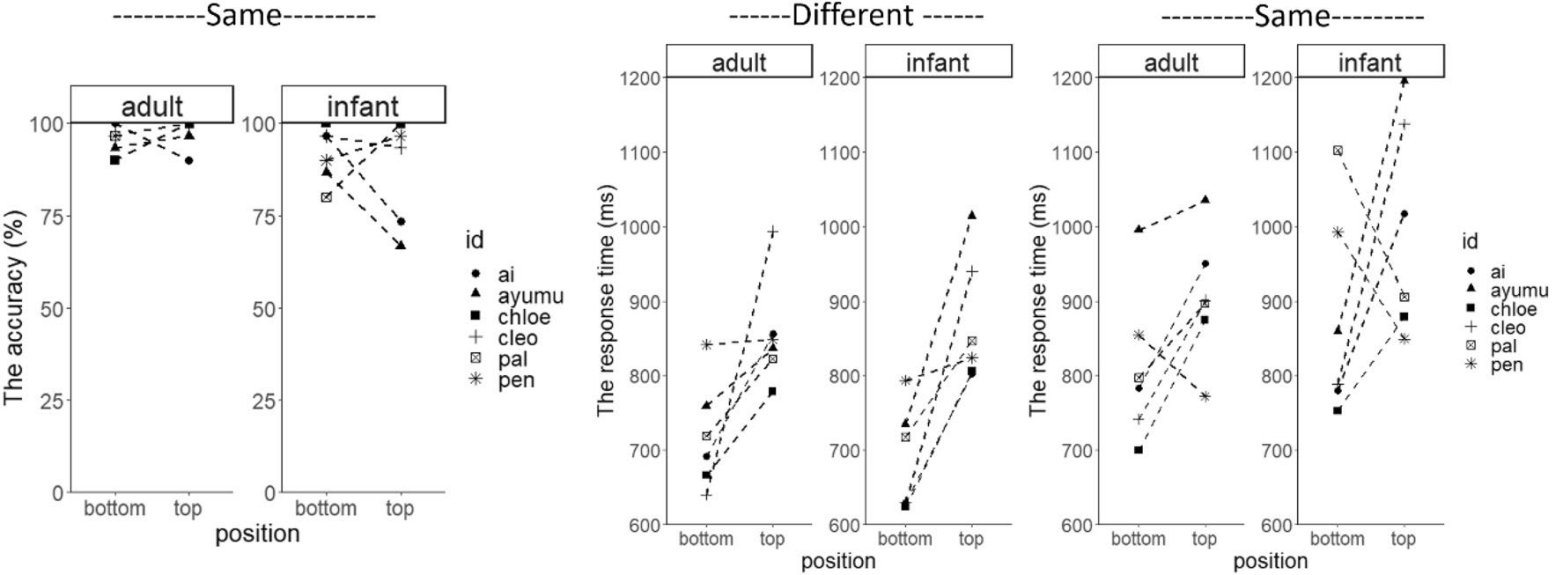
The average accuracy and the response time in Experiment 1 (vertical array)

The accuracy was almost perfect when the condition was different (average accuracy ± SD: 99.8 ± 0.5%) but slightly reduced in the same condition (93.5 ± 4.3%, Fig. 2). We analyzed the arcsine transformed accuracy by a repeated-measures ANOVA and found a significant main effect of the condition (*F*_1, 5_ = 20.08, *p* = 0.007, *η*^2^_*p*_ = 0.80), and an approached significant main effect of age (*F*_1, 5_ = 4.90, *p* = 0.08, *η*^2^_*p*_ = 0.50), and interactions between condition and age (*F*_1, 5_ = 4.90, *p* = 0.08, *η*^2^_*p*_ = 0.50). The other main effect and the interactions were not significant (all *ps* > 0.38). The post hoc analysis (adjusted using Shaffer’s procedure) indicated that the accuracy was greater in the different condition than the same condition when the stimulus was an adult (*F*_1, 5_ = 11.91, *p* = 0.02, *η*^2^_*p*_ = 0.70) and an infant (*F*_1, 5_ = 14.71, *p* = 0.01, *η*^2^_*p*_ = 0.75). The accuracy for adult stimuli compared with infant stimuli was slightly greater when the condition was the same (*F*_1, 5_ = 4.90, *p* = 0.08, *η*^2^_*p*_ = 0.50), but the performance was perfect for both stimuli types when the condition was different.

A repeated-measures ANOVA of the response time revealed a significant main effect of position (*F*_1, 5_ = 7.25, *p* = 0.04, *η*^2^_*p*_ = 0.59) (Fig. 2, see also Supplementary Information), condition (*F*_1, 5_ = 28.44, *p* = 0.003, *η*^2^_*p*_ = 0.85), and age (*F*_1, 5_ = 6.78, *p* = 0.05, *η*^2^_*p*_ = 0.58), and an approached significant interaction between condition and age (*F*_1, 5_ = 5.84, *p* = 0.06, *η*^2^_*p*_ = 0.54). The other interactions were not significant (all *ps* > 0.16). The post hoc analysis indicated that the response time in the same condition was greater than in the different condition when the stimuli were infants (*F*_1, 5_ = 71.96, *p* < 0.001, *η*^2^_*p*_ = 0.94), but this tendency was not robust when the stimuli were adults (*F*_1, 5_ = 4.05, *p* = 0.10, *η*^2^_*p*_ = 0.45). The response time was greater for infant stimuli than adult stimuli in the same condition (*F*_1, 5_ = 9.64, *p* = 0.03, *η*^2^_*p*_ = 0.66), but not in the different condition (*F*_1, 5_ = 0.16, *p* = 0.71, *η*^2^_*p*_ = 0.03).

These results indicated the following. First, the response time when the target was presented at the top of the monitor was consistently longer than when it was presented at the bottom (i.e., the effect of position). This probably occurred as touching the top part of the monitor was simply physically more demanding because of the touch panel’s structure. Second, differentiating between faces from the same age category was more difficult than differentiating between faces from different age categories (i.e., the effect of the condition). This suggests that the faces from the different age categories were perceptually more different from each other than those from within the same age category. Third, the chimpanzees took more time when the target was an infant than when it was an adult, especially when they needed to discriminate between two different infant faces (i.e., the interaction effect between age and condition). On the other hand, the results did not show a congruency effect between the target’s age and position (i.e., the interaction effect between age and position). Hence, there was no evidence of correspondence between vertical space and adult/infant faces. Although the sample size was quite small, visual inspection of demographic factors (i.e., sex and birth experience) did not find any systematic individual differences (see also Table 1 for participant information).

#### Experiment 2 (horizontal array)

**Fig. 3:**
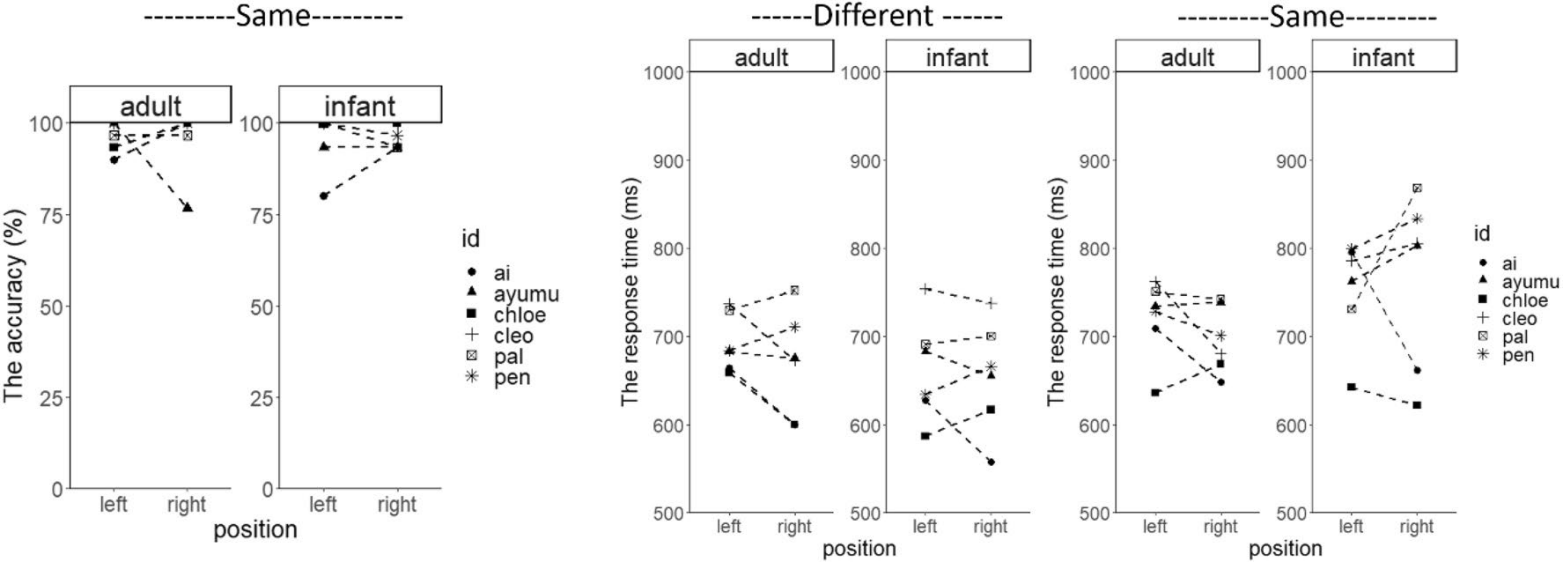
The accuracy and the average response time in Experiment 2 (horizontal array)

The accuracy was again almost perfect when the condition was different (99.1% ± 0.8%), but slightly reduced in the same condition (96.0 ± 3.8%, Fig. 3). We analyzed arcsine transformed accuracy by a repeated-measures ANOVA. We found no main effect or interactions was significant (all *ps* > 0.12).

A repeated-measures ANOVA of the response time revealed a significant main effect of the condition (*F*_1, 5_ = 27.47, *p* = 0.003, *η*^2^_*p*_ = 0.85), but not of position (*F*_1, 5_ = 0.35, *p* = 0.58, *η*^2^_*p*_ = 0.07) or age (*F*_1, 5_ = 1.74, *p* = 0.24, *η*^2^_*p*_ = 0.26, Fig. 3, see also Supplementary Information). The interaction between condition and age was significant (*F*_1, 5_ = 12.15, *p* = 0.02, *η*^2^_*p*_ = 0.71), but the other interactions were not (all *ps* > 0.17). The post hoc analysis indicated that the response time was greater for the same condition than for the different condition when the stimulus was an adult (*F*_1, 5_ = 11.43, *p* = 0.02, *η*^2^_*p*_ = 0.70) and an infant (*F*_1, 5_ = 22.48, *p* < 0.01, *η*^2^_*p*_ = 0.82). The response time was greater for infant stimuli than adult stimuli in the same condition (*F*_1, 5_ = 9.38, *p* = 0.03, *η*^2^_*p*_ = 0.65), but not in the different condition (*F*_1, 5_ = 2.45, *p* = 0.18, *η*^2^_*p*_ = 0.33).

As before, these results suggest that differentiating between faces from the same age category was more demanding than differentiating between faces from different categories (i.e., the effect of condition). Additionally, it took more time for the chimpanzees to discriminate between two different infant faces than in the other conditions (i.e., the interaction effect of age and condition). We did not find any effect of the position of the target, including the interaction between position and age. Therefore, there was no evidence of correspondence between horizontal space and adult/infant faces. When we look the results individually, the response time tended to be slightly shorter in adult-right and/or infant-left condition in many participants (see also Supplementary Information). It is noted that two individuals who show the opposite pattern (Ai and Chloe) were females who had birth experience, although it is difficult to conclude on it due to our small sample size.

### Image similarity analysis

**Fig. 4:**
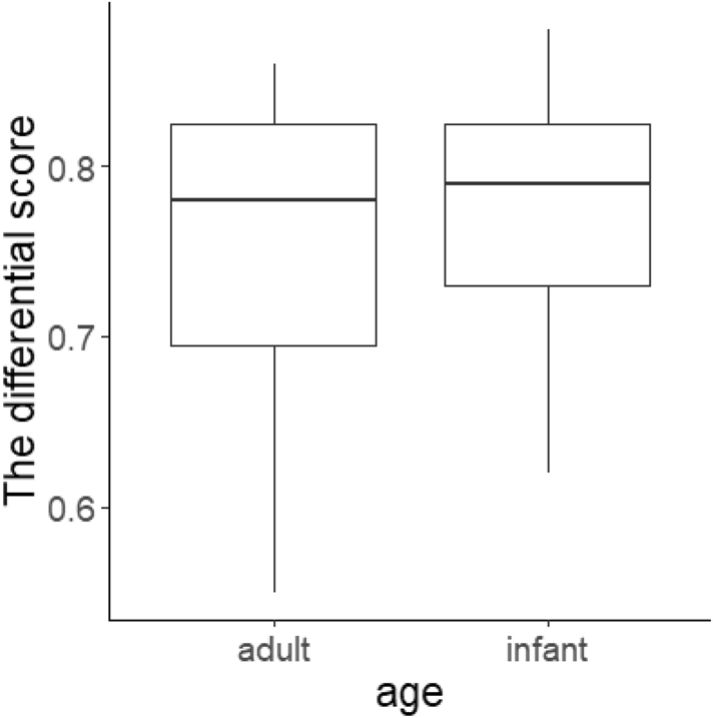
The differential score within each age category. The score was calculated based on the structural similarity index, and a greater mean value indicates that there is a larger difference between the stimuli. The statistical analysis found no significant difference between the adult and infant stimuli

Figure 4 illustrates the differential score between each stimulus within each age category, which was calculated based on the SSIM. If this value is 0 it means that the two images are the exactly same, while if it is greater it means that there is a larger difference between the stimuli. This differential score was compared using the Mann–Whitney *U* test. The results demonstrated that there was no difference between the average similarity of the adult and infant stimuli among the same age category (*U* = 106.5, *p* = 0.82). The findings indicate that the physical variation in the stimuli within each age category was not significantly different between the adult and infant faces in terms of low-level features. It is therefore unlikely that the reason for the chimpanzees’ asymmetric performance when differentiating between adult and infant faces is that the infant stimuli were more similar to each other than the adult stimuli.

## Discussions

The present study explored face processing related to age recognition from the aspect of a spatial mapping of face age in chimpanzees. The analysis of the performance and the response time indicated no effect of the position corresponding to the age category of the stimuli. That is, the results do not support the existence of the spatial representation of facial age in either a vertical (Experiment 1) or horizontal (Experiment 2) array in chimpanzees. The non-significant result of the correspondence between space and facial age implies some possibilities. First, there is a possibility that the variation of the results among the relatively small sample size (*n* = 6) may have masked the subtle effect, if any. This is because in the horizontal array (Experiment 2), there was a weak tendency, where the response time tended to be slightly shorter in adult-right and/or infant-left condition. Thus, a weak horizontal spatial mapping might exist in chimpanzees, but such a modest spatial association might not be robust to any artifacts (e.g., an individual’s position bias).

Second possibility is that chimpanzees may not recognize faces as “adult” or “infant” as we do; in other words, they may not extract conceptual age categories from faces. Previous studies have demonstrated that non-human primates also recognize a face image as representing faces by reporting the neural activities that are selective for faces (e.g., Tsao et al. 2003, 2008). Moreover, the present study indicates that the face discrimination performance differed between the same condition versus the different condition. This indicates that the faces across the different categories were perceptually more different than the faces within the same category for the chimpanzees. A previous study also demonstrates that chimpanzees can differentiate adult faces and infant faces (Kawaguchi et al. 2020). This evidence indicates that chimpanzees explicitly extracted shared visual features within each category. Therefore, the chimpanzees should have at least recognized that the stimuli we used were representing faces, which can be dissociated into two categories. However, that category may not have been based on age, but something else such as low-level features including the color difference.

The other possibility is that even though the chimpanzees extracted conceptual age category from face images, they may have not associated it with space for some reasons. As what we know about time recognition in non-human primates is quite limited and our study was explorative, it is difficult to conclude whether chimpanzees do not recognize the infant-adult in time sequence, or if they recognize it as related to time but do not associate time with space. Previous research has suggested that some time-related recognition in humans is shared with non-human primates. For example, mental time travel, in which past events are reconstructed, and the future is imagined, is partially shared with non-human primates (for review, Suddendorf and Corballis 2010). However, how similar their time recognition is to humans or whether they have concept of time is still unclear. This is because previous studies have focused specifically on the aspect related to decision-making based on episodic memory or future planning instead of testing a time concept itself. Therefore, how non-human primates comprehend time, especially longer time such as recognizing another individual across decades from their infancy to adulthood, should be examined further.

Another finding of the present study is that our chimpanzees had a faster response time when discriminating between adult faces than when discriminating between infant faces. These results did not occur because of the difference of physical similarities among the adult faces versus the infant faces, as the image analysis demonstrated that both were comparable. Human own-age bias is usually considered to reflect “more extensive, recent experiences with one’s own-age group relative to other-age groups” (Rhodes and Anastasi 2012, p.146). Similarly, this chimpanzees’ asymmetric efficiency in face processing probably arose because they were attuned to processing adult faces based on their daily face experiences. Our chimpanzees have experience of interacting with infants in the past, but they had not seen infants for a while. However, they were living socially and interacting with other adult group members in their daily life. These asymmetric amounts of experiences of adult and infant conspecifics have likely led to the current results. This is probably not specific to our chimpanzees but is likely more general. Given that chimpanzee adults generally have more interactions with adults than individuals belonging to different age categories, they likely have a superior face processing ability for adult than infant individuals.

These results are understandable in line with previous human studies that suggest the existence of the own-age bias. In our chimpanzees, extensive exposure to adult conspecific faces in their daily life has likely shaped their perceptual system toward expertise for adult faces. Nevertheless, infantile face coloration in chimpanzees may also be particularly responsible for the impaired discrimination performance for infant faces. Chimpanzee infant faces are different from adult faces, both in shape and color (Kawaguchi et al. 2020). Previous studies found that chimpanzees specifically pay attention to the conspicuous infant face coloration, which is a much paler color than adults (Kawaguchi et al. 2019a, 2020). Therefore, it is possible that the chimpanzees’ attention was attracted by the unfamiliar face color (i.e., infantile face color), and their fluent face processing was subsequently impaired. It is worth testing which particular facial feature causes impaired face processing for infant faces in chimpanzees.

The present study has some limitations. First, it is challenging to interpret the null result of spatial mapping of face age only from the present study. As mentioned earlier, some possibilities remain. We can tell from the results that the positive evidence that chimpanzees were extracting the age concept from faces was not found, yet we cannot fully deny that possibility. However, given that recognition of age concept in non-human primates has been seldom studied, the result can be a stepping stone for future comparative cognitive studies of age or time recognition, including mental timelines. On the other hand, we found that chimpanzees show asymmetric performance for discriminating between adult faces and infant faces, which is seemingly similar to human own-age bias. Nevertheless, we cannot conclude that the efficient face processing for adult faces in our chimpanzees is the same phenomenon as own-age bias in humans. This is because it is unclear whether chimpanzees of other-age classes such as juveniles also have efficient face processing selectively for their cohort’s faces. To understand whether this bias in chimpanzees is identical to the own-age bias in humans, a future study needs to examine this issue using chimpanzees from a wider age range, both as participants and as stimuli.

In conclusion, the present study explored two dimensions of facial age recognition in chimpanzees: spatial mapping and the effect of the different amount of experience. The current data did not support the existence of spatial mapping of the age categories in chimpanzees. However, we found the evidence of the superior processing of adult faces compared to infant faces in adult chimpanzees. As far as we know, this is the first report of an asymmetric face processing efficiency between infant and adult faces in non-human primates. This finding revealed a new aspect of chimpanzee’s face recognition related to age, which is seemingly similar to that of humans.

## Supplementary Information

Below is the link to the electronic supplementary material.

Supplementary file1 (DOCX 194 kb)

Supplementary file2 (XLSX 156 kb)

## Data Availability

Raw data is available, and the stimuli are available upon a request.
