# Supplementary material for: No evidence of spatial representation of age, but “own-age bias” like face processing found in chimpanzees
Source: Anim Cogn. 2021 Oct 2;25(2):415–24. doi: 10.1007/s10071-021-01564-7 (PMC8940789; doi:10.1007/s10071-021-01564-7)
Supplement: Supplementary file 1 — Supplementary file1 (DOCX 194 kb) [file 10071_2021_1564_MOESM1_ESM.docx]

**No Evidence of Spatial Representation of Age, but “Own-Age Bias” like Face Processing Found in Chimpanzees**

Yuri Kawaguchi, Ikuma Adachi, Masaki Tomonaga


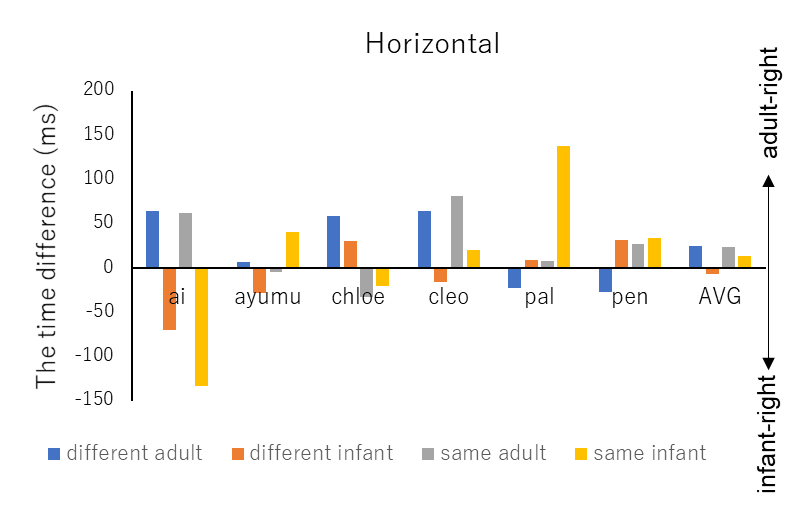

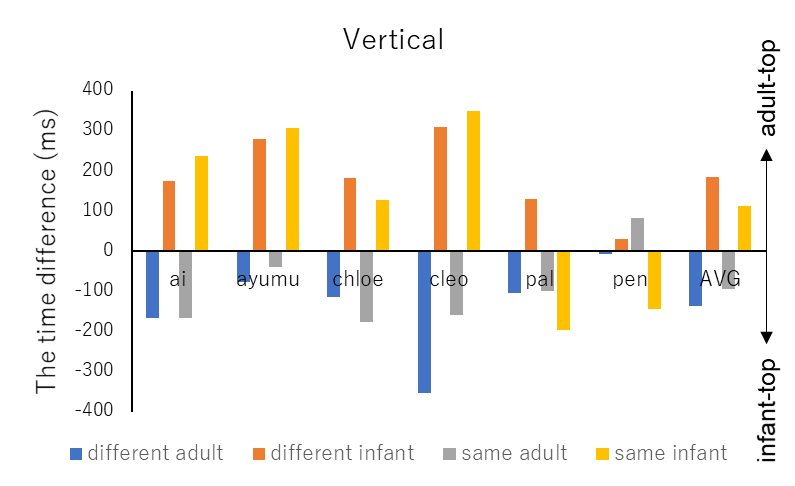


Figure A1. The individual and average (AVG) data of the response time difference between top and bottom in vertical (Experiment 1) and left and right in horizontal (Experiment 2) for each condition.
